# Reopening Oral Health Services during the COVID-19 Pandemic through a Knowledge Exchange Coalition

**DOI:** 10.1177/23800844211011985

**Published:** 2021-04-27

**Authors:** M. McNally, L. Rock, M. Gillis, S. Bryan, C. Boyd, F. Kraglund, B. Cleghorn

**Affiliations:** 1 Faculty of Dentistry, Dalhousie University, Halifax, NS, Canada; 2 School of Dental Hygiene, Dalhousie University, Halifax, NS, Canada; 3 Provincial Dental Board of Nova Scotia, Bedford, NS, Canada; 4 College of Dental Hygienists of Nova Scotia, Halifax, NS, Canada

**Keywords:** health knowledge attitudes practice, prevention and control, personal protective equipment, dentistry, practice guidelines, surveys

## Abstract

**Background:**

The COVID-19 novel coronavirus closed oral health care in Nova Scotia (NS) Canada in March 2020. Preparing for a phased reopening, a knowledge exchange coalition (representing government, academia, hospitals, oral health professions, and regulators) developed return-to-work (RTW) guidelines detailing the augmentation of standard practices to ensure safety for patients, oral health care providers (OHPs), and the community. Using online surveys, this study explored the influence of the RTW guidelines and related education on registered NS OHPs during a phased return to work.

**Methods:**

Dissemination of R2W guidelines included website or email communiques and interdisciplinary education webinars that coincided with 2 RTW phases approved by the government. Aligned with each phase, all registered dentists, dental hygienists, and dental assistants were invited to complete an online survey to gauge the influence of the coalition-sponsored education and RTW guidelines, confidence, preparedness, and personal protective equipment use before and after the pandemic.

**Results:**

Three coalition-sponsored multidisciplinary webinars hosted 3541 attendees prior to RTW. The response to survey 1 was 41% (881/2156) and to survey 2 was 26% (571/2177) of registrants. Survey 1 (82%) and survey 2 (89%) respondents “agreed/strongly agreed” that R2W guidelines were a primary source for guiding return to practice, and most were confident with education received and had the skills needed to effectively treat patients during the COVID-19 pandemic. Confidence and preparedness improved in survey 2. Gowns/lab coat use for aerosol-generating procedures increased from 26% to 93%, and the use of full face shields rose from 6% to 93% during the pandemic.

**Conclusions:**

A multistakeholder coalition was effective in establishing and communicating comprehensive guidelines and web-based education to ensure unified reintegration of oral health services in NS during a pandemic. This multiorganizational cooperation lay the foundation for responses to subsequent waves of COVID-19 and may serve as an example for collaboratively responding to future public health threats in other settings.

**Knowledge Transfer Statement:**

The return-to-work strategy that was developed, disseminated, and assessed through this COVID-19 knowledge exchange coalition will benefit oral health practitioners, professional regulators, government policy makers, and researchers in future pandemic planning.

## Background

Early in 2020, the onset of a novel coronavirus, COVID-19, resulted in a wave of transmission around the world. The person-to-person mode of transmission through respiratory droplets and aerosols (Harrel and Molinari 2004; Peng et al. 2020) risked compromising the safe delivery of oral health care. Closure of dental clinics soon followed in many regions. Oral health practitioners in Nova Scotia (NS), a small province on the east coast of Canada (population 979,000; Government of Canada SC, 2017), were ordered by the chief medical officer of health on March 20, 2020 (Government of Nova Scotia, 2020b), under its Health Protection Act, to close all dental clinics except for designated centers to provide emergency services (Provincial Dental Board of Nova Scotia, 2020a).

At the outset of the provincial closure, a collaboration evolved among regulators of oral health professions, oral health profession member organizations and practitioners, the Nova Scotia Department of Health and Wellness (NSDHW), dental educators, and researchers to address the complexities of shutting down services and determine standards and processes for the provision of interim emergency services and the eventual return to provision of routine care. The reopening plan consisted of 3 distinct phases: phase 1, emergency action plan (Provincial Dental Board of Nova Scotia, 2020a); phase 2, emergency and urgent care (Albert et al. 2020a); and phase 3, comprehensive care (Albert et al. 2020b).

The transmission of COVID-19 through respiratory droplets meant that aerosol-generating procedures (AGPs) used for routine dentistry and dental hygiene treatments posed a risk to oral health care providers (OHCPs) and patients (Harrel and Molinari 2004; Peng et al. 2020). It was generally accepted at this time that routine standard precautions for infection prevention and control (IPC) were likely insufficient (Meng et al. 2020). Standards and protocols to advance services beyond non-AGP emergency procedures were critical. Members of the collaboration described above developed return-to-work (RTW) guidelines augmenting standard practices to align with best available evidence and to meet provincial standards for approval. Guidelines were widely disseminated, and a series of open access education webinars was provided by infectious disease experts and contributors to the RTW guidelines (Provincial Dental Board of Nova Scotia, 2020b).

Stakeholders, particularly the Provincial Dental Board of Nova Scotia (PDBNS), regulator for registered dentists and dental assistants (RDAs), and the College of Dental Hygienists of Nova Scotia (CDHNS), regulator for registered dental hygienists (RDHs), recognized that OHCPs were influenced by the known risks for transmission associated with AGPs, critical research gaps on standards of IPC, and inconsistent messaging from national and international health and regulatory agencies (e.g., World Health Organization, United States Centers for Disease Control, and the Public Health Agency of Canada) regarding policies and procedures to protect providers and patients from COVID-19 infection. This raised concerns for regulators that were coupled with their mandate to forecast and address ever-changing oral health care service needs and OHCP safety considerations, particularly in preparing for future potential “waves” of COVID-19. As part of the NS RTW initiative, it was critical to explore the value of guidelines and education programming for dentists, RDAs, and RDHs returning to work. Through multiple surveys and frequent stakeholder knowledge exchange, this study explored the influence of evidence-based guidelines and related education on RTW practices of NS dentists, RDAs, and RDHs during a phased RTW plan. The purpose of this work is to report baseline data on the surveys at 2 critical RTW phases and to explore whether the integrated provincial multistakeholder approach was effective in establishing and communicating comprehensive guidelines to ensure a successful reintegration of oral health services in NS following the initial COVID-19 provincial shut down.

## Methods

RTW phase 2 (June 5, 2020; Albert et al. 2020a) and phase 3 (June 19, 2020; Albert et al. 2020b) were approved by the NSDHW. Dissemination of RTW guidelines included frequent website or email communiques and interdisciplinary education webinars. Two voluntary, anonymous surveys were developed to coincide with RTW phases 2 and 3 to gauge confidence and perceived preparedness of dentists, RDAs, and RDHs returning to work.

Survey questions detailed in Appendix Table 1 were modified from existing surveys (Gerbert 1987; Goulia et al. 2010; Naghavi et al. 2012; Ramich et al. 2017; Dost et al. 2020; Jin et al. 2020) and in response to regulator and stakeholder input. Demographic questions (Table 1) documented participants’ gender, profession, nature of employment, and number of dependents. Five-point Likert scale questions (*strongly agree* to *strongly disagree*) were organized under 6 domains: perceived risks, workplace preparedness, individual preparedness for returning to practice, financial concerns during the pandemic, confidence in IPC protocols and personal protective equipment (PPE) effectiveness and availability, and IPC protocols and PPE use (Appendix Table 1). Multiple-response questions explored information sources and PPE choices for non-AGPs and AGPs before and during the pandemic. Questions were vetted with stakeholders for content and face validity. SurveyMonkey provided the online platform for the anonymous surveys.

**Table 1.** Responses to Demographic Questions.

	Survey 1 (*n* = 881)		Survey 2 (*n* = 571)	
Variable	*n*	%	*n*	%
Age, y				
<20	1	0.1	1	0.2
20–29	119	13.6	61	10.7
30–39	202	23.0	112	19.7
40–49	234	26.7	141	24.8
50+	322	36.7	253	44.5
No response	3		3	
Gender				
Female	738	83.8	474	83.3
Male	134	15.2	93	16.3
Other gender identity	1	0.1	0	0.0
Prefer not to say	8	0.9	2	0.4
No response	0		2	
Number of children				
0	266	30.2	157	27.5
1	146	16.6	111	19.5
2	328	37.3	204	35.8
3	100	11.4	70	12.3
4 or more	40	4.5	28	4.9
No response	1		1	
Care for elder/dependent family member				
No	750	85.4	492	86.3
Yes	128	14.6	78	13.7
No response	3		1	
Type of employee				
Associate	70	7.9	52	9.1
Employee	621	70.5	384	67.3
Other	24	2.7	13	2.3
Practice owner	166	18.8	122	21.4
Profession				
Dentist	246	28.0	182	31.9
Dental assistant	363	41.3	253	44.4
Dental hygienist	270	30.7	135	23.7
No response	2		1	
Participated in both surveys				
No	NA		53	9.3
Yes	NA		517	90.7
No response			1	

Registered dentists, RDAs, and RDHs were invited to participate via an online link circulated by regulators. Survey 1 opened June 5, 2020, to coincide with phase 2 reopening of clinics for emergency and urgent care. Survey 2 aligned with June 19, 2020, phase 3 reopening of clinics for comprehensive oral health care. Surveys were available for 15 d.

The program evaluation and quality improvement aim of the surveys did not require institutional ethics review. However, ethical standards for informed consent and confidentiality were instantiated within the study. Invitees were apprised that participating in the survey was voluntary and anonymous, and they were provided details regarding the purpose of the survey, the nature of questions, and use and security of responses. Consent was confirmed by the participant choosing to open the survey link.

Data were analyzed using SPSS version 25.0 software (IBM Corp). Threshold for significance was set at *P* < 0.05, and all statistical tests were 2-tailed. Missing data were handled though a complete-case analysis method. A descriptive analysis was performed to report absolute and relative frequencies of responses to each question. A comparison of survey responses and type of oral health profession was assessed using a chi-square analysis (or a Fisher exact test when more than 20% of cells contained expected frequencies of <5). Responses to questions in survey 1 and survey 2 were compared using a McNemar test. A comparison of the use of PPE and IPC before and during the pandemic was performed using a 2-sample *Z* test.

## Results and Discussion

Province-wide education webinars (May 29 and 30, June 17) hosted 3541 attendees. The overall response to survey 1 was 41% (881/2156), representing 44% (246/560) of dentists, 45% (363/800) of RDAs, and 34% (270/796) of RDHs. Response to survey 2 was 26% (571/2177), representing 32% (182/571) of dentists, 32% (253/802) of RDAs, and 17% (135/804) of RDHs. Although 310 fewer participants completed survey 2, 91% had also participated in survey 1 indicating strong engagement.

### Demographic Information

Demographic responses (Table 1) for 881 participants in survey 1 indicate that 28% were dentists, 41% registered RDAs, and 31% registered RDHs. Respondents were primarily employees (70%) followed by practice owners (19%). Of practice owners, 162 were dentists and 4 were RDHs. Of the 571 respondents for survey 2, 32% were dentists, 44% were RDAs, and 24% were RDHs. Respondents were primarily employees (67%) followed by practice owners (21%).

### Perceived Risks

Most respondents perceived that returning to work would increase their risk for contracting COVID-19, with 75% (survey 1) and 73.5% (survey 2) either agreeing/strongly agreeing with this statement (Appendix Table 2). Only 12.5% (survey 1) and 14% (survey 2) disagreed/strongly disagreed that an increased risk due to dental procedures would accompany their RTW. Similarly, 71% of survey 1 and 69% of survey 2 respondents considered their families to be at higher risk with their return to work.

RDAs (84% survey 1, 79% survey 2) and RDHs (88% survey 1, 89% survey 2) perceived more risk to themselves than did dentists (49% survey 1, 55% survey 2). At the time of survey 1, RDAs and RDHs were more likely to perceive themselves to be at risk as compared with dentists (odds ratio [OR]: 6.25; 95% confidence interval [CI] 4.5–8.7, *P* < 0.001). Likewise, RDAs and RDHs perceived more risk to their families than did dentists in both survey 1 (OR: 5.1; 95% CI 3.7–7.0, *P <* 0.001) and survey 2 (OR: 3.2; 95% CI 2.2–4.7, *P* < 0.001). Because a significantly higher proportion of RDAs and RDHs identified as female (99% and 97% respectively) as compared with dentists (48%), gender was assessed for collinearity and was ruled out as a confounding factor.

Despite the overall perception of increased risk to themselves and those close to them, only 39% of survey 1 respondents perceived that friends and family would distance themselves after the respondent returned to work. This proportion dropped significantly (to 16%) in survey 2 (*P* < 0.001). There were significant differences between professions regarding distancing of family and friends. In survey 1, 16% of dentists expected family and friends to distance themselves upon the dentist’s return to work, compared with 48% of RDAs and 50% of RDHs (OR: 4.7, 95% CI 3.3–7.0; *P <* 0.001). In survey 2, 8.5% of dentists reported actual distancing of their family and friends, as compared to 21% of RDAs and 17% of RDHs (OR: 2.7, 95% CI 1.5–4.8; *P* < 0.001).

**Figure 1.**
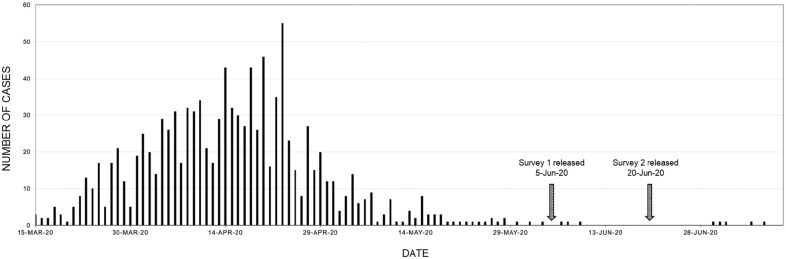
Daily COVID-19 cases reported in Nova Scotia from March 15, 2020, to July 11, 2020 (Government of Nova Scotia, 2020a).

One-third of respondents (33%) in survey 1 anticipated that patients would be reluctant to continue in their care because of high risk. Yet only 15% of survey 2 respondents agreed/strongly agreed that there was a decrease in patients requesting routine appointments once comprehensive dental care reopened (63% disagreed). Several possible explanations for this positive trend include care needs that were not addressed during the shutdown, a desire to address routine and elective care needs before a “second wave,” and the public’s confidence in established IPC protocols. Patient confidence in seeking care could also be attributed to the low number of cases (1066 since the first reported case on March 15, 2020) of community spread in NS at that time (Fig. 1; Government of Nova Scotia, 2020a).

Most RDAs (64%) and RDHs (68%) reported that patients had expressed concerns about COVID-19 in the dental office in survey 2. Fewer than half (45%) of dentists reported the same (OR: 2.3, 95% CI 1.6–3.3; *P* < 0.001). Most (85%) survey 2 respondents reported fewer escorts, parents, and guardians in the operatory. These results were affirming to provincial oral health profession regulators who expressly promoted the importance of minimizing unnecessary attendees in dental operatories.

### Workplace Preparedness

Of survey 1 respondents, 82% considered the RTW guidelines for phase 2, emergency and urgent care, helpful in guiding their return to work (Appendix Table 3). This increased to 89% in survey 2. Only 7% (survey 1) and 2% (survey 2) disagreed. All 3 professional groups had high rates of agreement with this statement (75% or higher), with dentists having the highest agreement (92% in survey 1 and 96% in survey 2).

Overall, 88% of survey 1 respondents agreed/strongly agreed that these guidelines were used by their workplace to develop a phase 2 site-specific reopening plan. This percentage was higher for dentists (97%) than for RDAs (84%) and RDHs (85%; *P* < 0.001). Just more than 93% of individuals who completed survey 1 agreed that protocols and guidelines had been developed at their workplace for screening and managing suspected/confirmed COVID-19 patients. Here, again, dentists reported higher rates of agreement with this statement (98%) than RDAs (93%) and RDHs (90%) did (*P* = 0.002). However, by survey 2, all 3 groups reported similarly high rates of agreement (97%, 94%, and 97%, respectively) with this statement (*P* = 0.28). In addition, in survey 2, respondents were asked whether their workplace had protocols in place for prescreening for COVID-19 symptoms during appointment scheduling (96% agreed/strongly agreed) and for rescreening before entry into the clinic (93% agreed/strongly agreed).

At survey 2, 81% of dentists agreed/strongly agreed that initial preparations at their workplace were adequate to prevent the spread of COVID-19. Significantly fewer RDAs (62%) and RDHs (65%) agreed/strongly agreed with this statement (OR: 2.5, 95% CI 1.6–3.8; *P* < 0.001).

### Individual Preparedness for Returning to Practice

A high proportion of respondents reported comfort with their understanding of COVID-19 symptoms and risk at both surveys (93.5%), with slight differences among professions. Survey 1 (91%) and survey 2 (94%) respondents strongly agreed/agreed they were comfortable with their understanding of safety and IPC protocols (Appendix Table 4). Dentists’ comfort with their understanding of protocols (99% strongly agreed/agreed for both surveys) was significantly higher than RDAs or RDHs who reported, respectively, 87% and 88% for survey 1 (OR: 11.6, 95% CI 3.6–37.0; *P* < 0.001) and 92% and 88% for survey 2 (OR: 7.5, 95% CI 1.8–31.9; *P* = 0.001).

Most respondents agreed they were comfortable with their training and education on COVID-19 safety and IPC protocols (76% in survey 1 and 86% in survey 2). Dentists were more likely to agree with this statement than the other professions: 94% (survey 1) compared with 69% RDAs and 69% for RDHs (OR: 6.8; 95% CI 4.0–11.9; *P* < 0.001). Comfort with education and training remained constant between survey 1 and survey 2 (*P* = 0.408). However, it significantly increased for RDAs and RDHs from survey 1 to survey 2 (*P* = 0.014 and *P = 0.003*, respectively). Given employment layoffs during the initial shutdown, it is likely that more training occurred following phase 2 return to the workplace (i.e., between surveys).

Survey 1 (85%) and survey 2 (87%) respondents strongly agreed/agreed they had the skills needed to effectively treat patients during the COVID-19 pandemic. Of dentists, 98% in survey 1 and 93% in survey 2 strongly agreed/agreed that they had the necessary skills. RDAs reported strongly agreeing/agreeing at 81% for survey 1 and 84% for survey 2, and RDHs strongly agreed/agreed at 80% for survey 1 and 84% for survey 2. Dentists’ rate of agreement was significantly higher than that of RDAs and RDHs in both surveys (OR: 24.8, 95% CI 9.9–61.8; *P* < 0.001 [study 1] and OR: 2.7, 95% CI 1.4–5.1; *P* = 0.033 [study 2]).

Most OHCPs (84% of survey 1 and 83% of survey 2 respondents) strongly agreed/agreed that they would get a vaccine for COVID-19 if and when one became available. Approximately 90% of dentists in both surveys agreed they would be vaccinated, but significantly fewer RDAs (79% for survey 1 and 78% for survey 2) and RDHs (85% for survey 1 and 81% for survey 2) strongly agreed/agreed they would be vaccinated. Dentists were >2½ times more likely to report accepting a vaccine than RDAs were (OR 2.7, 95% CI 1.5–4.8; *P* < 0.001) and twice as likely as RDHs were (OR 2.2, 95% CI 1.1–4.3; *P* = 0.02).

### Financial Concerns about Practicing during the Pandemic

Both surveys assessed financial concerns associated with returning to practice (Appendix Table 5). Dentists were more likely to acknowledge a significant financial burden to their office/workplace than RDAs and RDHs were, both in survey 1 (89% vs. 60% and 70%, respectively) and in survey 2 (72% vs. 50.5% and 63%). In survey 1, 71.5% of all OHCPs agreed that the requirements for enhanced standard precautions when performing AGPs would be a significant financial burden to their office/workplace. This dropped to 61% by survey 2 (OR: 1.6, 95% 1.3–2.0; *P* = 0.003), which suggests that the financial impact of enhanced precautions may have been somewhat less than anticipated. It could also be reflective of government-funded PPE made available at no cost to oral health providers, a program that was in effect at the time of phase 3 RTW.

Dentists reported much higher rates of agreement that enhanced standard precautions caused a significant personal financial burden than did RDAs and RDHs, both in survey 1 (78% vs. 20% and 22%, respectively; *P* < 0.001) and survey 2 (63% vs. 11% and 6.5%, respectively; *P* < 0.001). Over the 2 surveys, a total of 155 RDAs and RDHs skipped this question or selected “not applicable,” compared with only 17 dentists. A significantly higher proportion of dentists (76%) reported that their personal income had been reduced since returning to work during the COVID-19 pandemic compared with RDAs and RDHs (OR: 14.7; 95% CI 9.4–22.9; *P* < 0.001).

In survey 2, 35% of respondents agreed the added cost of enhanced standard precautions affected their PPE choices, with dentists (44%) more likely to indicate agreement than RDAs (33%) or RDHs (27%; *P* < 0.001). Nearly 40% of respondents in survey 2 indicated that the additional cost of enhanced standard precautions was being passed along to patients. RDHs (47.5%) were more likely to indicate this than dentists (36%) or RDAs (38%) were (*P* < 0.001).

### Confidence in IPC Protocols and PPE Effectiveness and Availability

Most dentists in survey 2 agreed that they were confident that the COVID-19 safety and IPC protocols protect OHCPs (91%) and patients (96%) from COVID-19 (Appendix Table 6). However, RDAs and RDHs had significantly lower rates of agreement, particularly regarding protection of OHCPs (59% and 58% agreement, respectively; OR: 6.8, 95% CI 4.0–11.7; *P* < 0.001). Lower confidence of IPC protocol effectiveness experienced by some OHCPs could be the reflective of concerns described above regarding uncertainty around transmission modes and inconsistent prevention strategies publicized in the early days of the pandemic.

In survey 1, fewer than half (41%) of the OHCPs agreed that there would be adequate supply of PPE once they returned to work, 30% disagreed, and 29% were neutral. RDAs were more likely to agree (47%) than dentists (42%) or RDHs (34; *P* < 0.004). Survey 2 found 52.5% of OHCPs agreed their preferred PPE items were available for purchase when needed, whereas 29% disagreed. There were no significant differences among the professions (*P* = 0.175). Most survey 2 respondents (65%) agreed that the availability of specific products influenced their choice of PPE; dentists were more likely to agree (75%) than were RDAs (56%) and RDHs (67%; *P* < 0.001).

### IPC Protocols and PPE Use Practices

This domain was covered only in survey 2 to determine OHCP IPC protocols and their use of PPE before and during the pandemic (Appendix Table 7). The results clearly show changes in PPE practices during the COVID-19 pandemic. Before the pandemic, most OHCPs of all groups did not wear a mask preoperatively or when taking a medical history (dentists 67%, RDAs 57%, and RDHs 81%). During the pandemic, 98% of dentists, 96% of RDAs, and 95% of RDHs indicated they always wear a mask preoperatively.

Sixty-three percent of respondents strongly agreed/agreed that they knew and strictly followed PPE donning and doffing procedures before the pandemic. Dentists reported lower rates of agreement (54.5% donning; 56% doffing) prepandemic than did RDAs (69% donning; 67% doffing) and RDHs (64% donning; 68% doffing). During the pandemic, 97% of all OHCPs indicated that they knew and strictly followed PPE donning (dentists 98%, RDAs 96%, RDHs 98.5%) and doffing (dentists 98%, RDAs 96%, RDHs 97%) procedures. Dentists (98%) strongly agreed/agreed that their coworkers knew the correct PPE to wear and strictly followed correct procedures to don and doff; RDAs and RDHs were less certain (85% and 78% respectively). Interestingly, only 1% of dentists, 8% of RDAs, and 12% of RDHs disagreed/strongly disagreed.

NS practices were not required to make major infrastructure changes, such as air filtration upgrades or changes to existing office designs to enclose operatories, nor were they required to observe air changes per hour “settling times.” Survey 2 documented that 63% of OHCPs did not restrict AGPs to enclosed operatories only, and 65% did not observe settling times after AGPs before operatory disinfection.

### Information Sources

**Figure 2.**
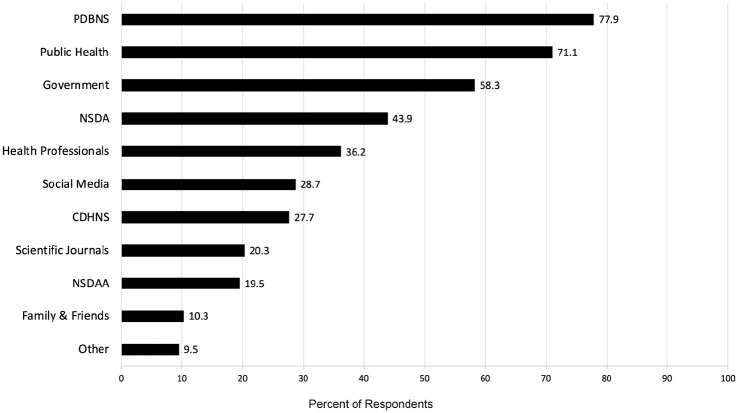
Survey 1 responses indicating participants’ main sources of information regarding COVID-19. The Provincial Dental Board of Nova Scotia (PDBNS) was the main overall source of information followed by public health announcements, government, Nova Scotia Dental Association (NSDA), health professionals, social media, College of Dental Hygienists of Nova Scotia (CDHNS), scientific journals, Nova Scotia Dental Assisting Association (NSDAA), family and friends, and other sources.

Figure 2 summarizes participants’ sources of information regarding COVID-19; 78% indicated they obtained information from the PDBNS resources and 71% from public health announcements. The CDHNS was the most cited resource for RDHs (89% of respondents).

### PPE Use before and after the Pandemic

**Figure 3.**
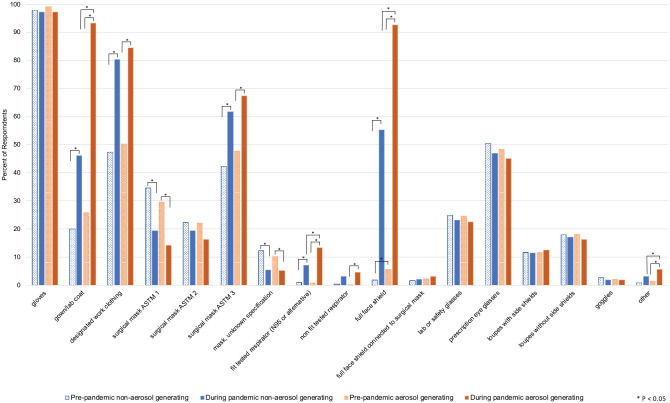
Summary of survey 2 respondents’ personal protection equipment (PPE) and infection prevention and control (IPC) practices before and during the COVID-19 pandemic.

Figure 3 illustrates survey 2 responses to summarize changes in PPE and IPC practices for AGPs and non-AGPs before and during the pandemic. The use of gowns or lab coats during AGPs during the pandemic increased from 26% to 93%. During the pandemic, 84% of OHCPs wore designated work clothing at the workplace, changing into “street clothes” when leaving the practice. A total of 50% of respondents indicated following this practice before the pandemic. Full face shields for AGPs rose from 6% to 93% during the pandemic. The use of N95 respirators during AGPs increased from less than 1% to 13%.

In summary, this study attempted a census sample, versus convenience or probability sampling, resulting in very high response rates free from selection bias and with a low margin of error for both survey 1 (2.54) and survey 2 (3.52). Survey fatigue of less than 50% (35.2%) between the 2 surveys, although acceptable, was not surprising given the frequency of competing requests for registrant input, feedback, and webinar participation among the professions. The preoccupation and time constraints brought on by the demands of having returned to work after a 10-wk absence also likely contributed to lower participation on the second survey. Volunteer bias was mitigated by the large response rate and low margin of error and response bias were mitigated by wording questions with a mixture of positive and negative responses. Studies seeking self-report data inherently risk recall bias and closed questions leave little opportunity for respondents to elaborate on reasons for responses. However, the overall interpretation of findings was enhanced by the input of regulators and by team members and contributors who are also clinicians involved in the RTW lived experience.

Table 2 summarizes studies published during the first 10 mo of the pandemic that explored the work experiences of OHCPs using similar parameters to our study. Twenty-four surveys queried perceived risks, knowledge confidence in, and implementation of COVID-19–specific IPC and PPE protocols, financial impacts, information sources, and/or education received. These studies were contextually different from ours because of differing aims, timing of questionnaires, timing of pandemic peaks in different locations, and evolving guidelines. Unlike NS, many jurisdictions did not have mandated practice closures.

**Table 2.** Summary of Return to Work Survey Studies.

First Author, Publication Date, Location(s)	Sample (*N* = Participants) (%)	Survey Date, Survey Extent	Subject Domains Queried
Ahmadi December 3, 2020 Iran	*N* = 240 dentists (89.2) general (10.8) specialist	June 10–25, 2020 51 questions	Demographics, awareness, trust and implementation of pandemic IPC protocols, availability/cost of PPE, personal financial impact, effects on personal life and financial status, attitudes/views on changes to practice management, services provided, quality of dental care for patients, continuation of dental care during pandemic, anxiety
Ahmed April 19, 2020 Multinational 30 countries	*N* = 650 dentists	March 10–17, 2020 22 questions	Anxiety of being infected and infecting family, awareness and implementation of CDC or WHO IPC COVID-19 guidelines; specific IPC/PPE practices not investigated
Ahmed May 2020 Pakistan	*N* = 810 (49) dentists (27) physicians (24) techs, DHs, nurses, DAs	February–March 20 31 questions	Demographics, knowledge of COVID-19, awareness and implementation of CDC guidelines, general IPC practices (not specific to dentistry) during “patient contact,” training attendance
Al-Khalifa August 19, 2020 Saudi Arabia	*N* = 287 dentists (61.7) general (38.3) specialist	May 23–31, 2020 26 questions	Demographics, adherence to Ministry of Health guidelines, perception of the COVID-19 pandemic, confidence in IPC measures, IPC/PPE practices before and after pandemic, preparedness and training/COVID-19 education received
Almas December 7, 2020 Pakistan	N= 343 dentists (58.6) specialist (41.4) general	Apr-May 20 22 questions	Demographics, IPC measures practiced, patient screening and management, comfort treating COVID-free patients and COVID-19 positive patients, following CDC, WHO and ADA clinical guidelines, attending COVID-19 seminar, financial impacts
Bakaeen September 30, 2020 Multinational 49 countries	*N* = 1251 dentists (63.9) general (36.1) specialist	March 28–April 10, 2020 25 questions	Demographics, comfort with preventive measures and provision of treatment, understanding of benefits and use of N95 respirators, PPE availability, attitudes toward treating COVID-19–positive or suspect patients, affect outbreak had on the workplace, financial impacts
Bellini September 22, 2020 Italy	*N* =1109 dentists	April 2–29, 2020 40 questions	Demographics, pre- and postpandemic PPE use and availability, pre- and postpandemic IPC practices, staff training, dental association usefulness as information source, risk perceptions, anxiety
Bontà October 31, 2020 Italy	*N* = 2788 dental hygienists	May 12–23, 2020 18 questions	Demographics, IPC measures (before patient arrival, in waiting room, in operatory) and PPE used, COVID-19 course received, risk perceptions
Cagetti June 1, 2020 Italy	*N* = 3599 dentists	April 10–17, 2020 17 questions	Demographics, IPC measures (before patient arrival, in waiting room, in operatory) and PPE used, COVID-19 course received, risk perceptions
Chaudhary July 23, 2020 Pakistan	*N* = 583 clinical: dentists, DAs/DHs Nonclinical: dental lab technician, cleaners, admin	Mar–June 2020 40 questions	Demographic, risk of exposure and fear of getting infected, concerns about colleagues, risk to and perceptions of family/friends, being avoided because of the job, anxiety, workplace preparedness, IPC training received; data shown for clinical and nonclinical groups separately and together; data for clinicians combined, not available by profession
Consolo May 15, 2020 Italy	*N* = 356 dentists	April 2–21, 2020 40 questions	Generalized Anxiety Disorder–7 test (GAD-7), risk of COVID-19 infection, PPE/IPC practices pre- and postlockdown, usefulness of professional associations for information, guidance and economic relief, training sessions in workplace, risk perceptions
De Stefani May 8, 2020 Italy	*N* = 1500 dentists (83.8) other (16.2) orthodontist	April 11–18, 2020 29 questions	Demographics, knowledge about COVID-19 infection transmission modalities and symptoms, comfort treating potentially infected patients, individual training and preparedness, information sources, risk perception
Duruk May 13, 2020 Turkey	*N* = 1958 dentists (65) general (35) specialist	March 16–20, 2020 23 questions	Demographics, risk perception for operators and family, PPE/IPC practices, information sources (Ministry of Health, professional organizations, social media), COVID-19 information session attendance
Gambarini June 1, 2020 Italy	*N* = 500 dentists	April 2020 8 questions	Demographics, risk to operators and patients, risk of aerosol transmission, modification of treatment and procedure protocols
Kamate March 31, 2020 Multinational	*N* = 860 dentists	December 25, 2019–February 20, 2020 24 questions	Demographics, information sources, knowledge, risk perception, informing staff of WHO guidelines, general adoption of IPC protocols
Martina June 18, 2020 Italy	*N* = 349 dentists (52.4) orthodontists (47.6) other	May 1–6, 2020 31 questions	Demographics, COVID-19 symptoms, perceived risk for operators during ortho procedures, care interruption for orthodontic and TMD patients, anxiety, fears of infection affecting health, family, income, social isolation, and continuity of treatment
Martinho and Griffin October 12, 2020 (preprint) United States	*N* = 454 endodontists	June–July 2020 24 questions	Demographics, COVID-19 knowledge, IPC/PPE practices, IPC/PPE effectiveness, perceived risks to operators, risk perception for operators, staff and family, patient hesitancy, treatments performed
Mustafa December 3, 2020 Saudi Arabia	*N* = 269 dentists	March 17–April 3, 2020 26 questions	Demographics, IPC training attended, COVID-19 symptom knowledge, attitude, risk perception, IPC measures, comfort managing COVID-19–positive patients and Ministry of Health dental public health policy support; specific PPE practices not investigated
Sarfaraz July 25, 2020 Multinational 23 countries	*N* = 385 dentists	May 14–20, 2020 17 questions	Demographics, knowledge of disinfection of surfaces and hands, risk perception during dentistry, effectiveness of disinfection guidelines; specific PPE practices not investigated
Singh Gambhir May 6, 2020 India	*N* = 215 dentists Private	March 2020 19 questions	Demographics, personal and clinic hygiene practices, knowledge/awareness of COVID-19 symptoms and transmission, medical treatment, and IPC/PPE guidelines; specific PPE practices not investigated
Sinjari August 10, 2020 Italy	*N* = 440 dentists	April 17, 2020 45 questions	Types of urgencies, treatments performed, IPC/PPE practices before and during pandemic, PPE costs, risk perception for infection, financial concerns, support by health and professional membership organizations
Stangvaltaite-Mouhat August 12, 2020 Norway	*N* = 1237 OHCPs (48) general and specialist dentists (19) DHs (33) DAs	May 4–Jun 26, 2020 Extent varied according to clinic policy	Demographics, practice management, additional IPC/PPE practices and training received, perception of risk and workplace preparation, COVID-19 information availability and sources, psychological impact; responses grouped according to participants’ clinic policy for accepting or not accepting COVID-19–positive patients for treatment
Tysia˛c-Miśta June 30, 2020 Poland	*N* = 875 dentists	April 6–16, 2020 Extent unspecified	Demographics, continuation or suspension of dental practice during pandemic (e.g., access to PPE and special equipment, adaptability of office structure to design requirements), risk perception, anxiety, assistance by Polish Ministry of Health and Polish Dental Association
Vieira-Meyer September 10, 2020 Brazil	*N* = 4048 dentists (28.8) general (71.2) specialist	March 29–April 4, 2020 15 questions	Demographics, COVID-19 knowledge, confidence in IPC/PPE protocol effectiveness, continuation or suspension of dental practice during pandemic; specific IPC and PPE practices not investigated

CDC, Centers for Disease Control and Prevention; DA, dental assistants; DH, dental hygienist; IPC, infection prevention and control; PPE, personal protective equipment; TMD, temporomandibular joint dysfunction; WHO, World Health Organization.

Three surveys compared the PPE and IPC of dentists before and after the pandemic and found dentists modified (Bellini et al. 2020) or increased their PPE use (Al-Khalifa et al. 2020; Consolo et al 2020; Sinjari et al. 2020). Others explored information sources influencing clinicians, finding that OHCPs looked to professional associations or government for guidance (Ahmadi et al. 2020; Al-Khalifa et al. 2020; Bellini et al. 2020; De Stefani et al. 2020; Stangvaltaite-Mouhat et al. 2020) and online for information from the World Health Organization, Centers for Disease Control and Prevention, American Dental Association, medical websites, and/or social media (Duruk et al. 2020; Kamate et al. 2020).

Studies addressing training support or knowledge exchange between practitioner, professional regulators, and public health institutions mainly observed that communication and coordination needed improvement (De Stafani et al. 2020; Mustafa et al. 2020; Sarfaraz et al. 2020; Tysiąc-Miśta 2020; Vieira-Meyer et al. 2020).

Most studies surveyed only dentists; Bontà et al (2020) surveyed only dental hygienists, whereas 3 studies surveyed dentists, dental hygienists, and dental assistants (Ahmed et al. 2020; Chaudhary et al. 2020; Stangvaltaite-Mouhat et al. 2020). No studies compared responses among the 3 professions to expose differences in their pandemic experiences.

Our study appears to be the only one to include consecutive surveys before and after reopening, demonstrating that confidence in available education and resources improved across all 3 professions and was key to preparedness.

## Conclusion

The NS survey study reflects 3 unique characteristics: 1) the inclusion of the broader clinical team, namely, dentists, dental assistants, and dental hygienists within the same study; 2) the circulation of surveys at 2 critical RTW phases that provide insight both before and shortly into the RTW; and 3) the investigation is framed within an interprofessional and multistakeholder knowledge exchange network. The integrated provincial multistakeholder collaboration was effective in establishing and communicating comprehensive guidelines and web-based education to ensure successful and unified reintegration of oral health services in NS following the initial COVID-19 provincial shutdown and laid the foundation for responses to subsequent waves of COVID-19. This process may serve as an example for collaboratively responding to public health threats in other settings.

In the 8 mo following the phase 2 RTW, the leadership and communication processes that had developed among representatives of the NSDHW, regulators of the oral health professions and oral health professions member organizations have remained active, enabling consistent mobilization for real-time decision making and for planning next steps. Findings from this work have created a level of confidence, particularly among regulators, that registrants are engaged with, and informed by, government-sanctioned guidelines. Responses regarding financial burden, PPE, and IPC practices have helped to focus advocacy among the oral health professions for government-sponsored support to offset direct financial burdens as well as to ensure a ready supply of required PPE. Baseline findings are informing website resource content (eg, frequently asked questions) and follow-up survey initiatives and will contribute to ongoing updates of provincial IPC guidelines, particularly in the prospective adoption of COVID-19 legacy PPE and procedures tailored to AGP and non-AGP procedures. The dissemination of results to participants and stakeholders includes a summary report disseminated to NSDHW and posted to CDHNS and PDBNS websites, oral health professions newsletters, NS continuing education events, and one-on-one stakeholder debriefs.

## Author Contributions

M. McNally, contributed to conception, design, data acquisition, and interpretation, drafted and critically revised the manuscript; L. Rock, contributed to conception, design, data analysis, and interpretation, drafted and critically revised the manuscript; M. Gillis, S. Bryan, contributed to conception, data acquisition, and interpretation, critically revised the manuscript; C. Boyd, contributed to design and data interpretation, drafted and critically revised the manuscript; F. Kraglund, B. Cleghorn, contributed to conception, design, and data interpretation, critically revised the manuscript. All authors gave final approval and agree to be accountable for all aspects of the work.

## Supplemental Material

sj-pdf-1-jct-10.1177_23800844211011985 – Supplemental material for Reopening Oral Health Services during the COVID-19 Pandemic through a Knowledge Exchange Coalition Click here for additional data file. Supplemental material, sj-pdf-1-jct-10.1177_23800844211011985 for Reopening Oral Health Services during the COVID-19 Pandemic through a Knowledge Exchange Coalition by M. McNally, L. Rock, M. Gillis, S. Bryan, C. Boyd, F. Kraglund and B. Cleghorn in JDR Clinical & Translational Research
