# Supplementary material for: Reopening Oral Health Services during the COVID-19 Pandemic through a Knowledge Exchange Coalition
Source: JDR Clin Trans Res. 2021 Apr 27;6(3):279–90. doi: 10.1177/23800844211011985 (PMC8207488; doi:10.1177/23800844211011985)
Supplement: sj-pdf-1-jct-10.1177_23800844211011985 – Supplemental material for Reopening Oral Health Services during the COVID-19 Pandemic through a Knowledge Exchange Coalition [file sj-pdf-1-jct-10.1177_23800844211011985.pdf]

Appendix Table 1. Survey 1 and Survey 2 Questions.

Survey 1 Question Number	Survey 2 Question Number	Question
**Domain 1. Perceived risks upon returning to practice**		
7	8	Dental/DH/DA procedures will increase/have increased my risk for COVID-19 (Gerbert 1987, Goulia et al. 2010)
8	9	My family will be/is at greater risk because of my work. (Goulia et al. 2010).
9	10	Family/friends will/now distance themselves from me because of my work. (Goulia et al. 2010)
10	N/A	Patients will be reluctant to continue in my care due to the perceived “high-risk” environment created by dental procedures. (Gerbert 1987)
N/A	12	Though my office is open to provide comprehensive care, I notice a decrease in patient requests for routine treatment appointments. (Gerbert 1987)
N/A	11	I have heard patients express concerns about COVID19 in the dental office.
N/A	13	There is a reduced presence of escorts, parents and guardians in the operatory room.
12	N/A	Media and news coverage related to COVID-19 is stressful. (Jin et al. 2020)
**Domain 2. Workplace preparedness for returning to practice**		
13	N/A	The Emergency and Urgent Care “Return to Work Guidelines for the Oral Health Professions of Nova Scotia” (released May 31st) are been helpful in guiding return to practice.
N/A	14	“Return to Work Guidelines for the Oral Health Professions of Nova Scotia: Phase/Stage 3” has been helpful in guiding return to practicing comprehensive care. (Hall et al. 2020b)
14	N/A	My workplace has developed its site-specific reopening plan based on the Emergency and Urgent Care “Return to Work Guidelines for the Oral Health Professions of Nova Scotia” (released May 31st). (Hall et al. 2020a)
15	15	Protocols and guidelines for screening and managing suspected/confirmed COVID-19 patients have been developed at my workplace. (Dost et al. 2020)

Survey 1 Question Number	Survey 2 Question Number	Question
N/A	18	The initial preparation at my workplace was enough to prevent the spread of COVID-19. (Goulia et al. 2010)
N/A	16	Before scheduling appointments in my workplace, patients are pre-screened using remote communications for COVID-19 symptoms, risk factors, underlying medical risk factors and the nature of the chief complaint
N/A	17	Before allowing patients entry into the clinic, staff rescreen them for COVID-19 symptoms, risk factors, underlying medical risk factors and the nature of the chief complaint.
**Domain 3. Personal preparedness for returning to practice**		
16	19	I am comfortable with my understanding of COVID-19 symptoms and risk factors.
18	20	I am comfortable with the education and training I have received on COVID-19 safety and infection control protocols.
17	21	I am comfortable with my understanding of safety and infection control protocols.
19	22	I have the skills I need to effectively treat patients during the COVID-19 pandemic. (Gerbert 1987)
11	26	I will get a vaccine to protect against COVID-19 when one becomes available. (Ramich et al. 2017)
**Domain 4. Financial concerns about practicing during COVID-19 pandemic**		
N/A	29	The additional cost of enhanced standard precautions when performing aerosol generating procedures influences my choice of PPE.
21	30	Requirements for enhanced standard precautions when performing aerosol generating procedures will be/has been a significant financial burden to my office/workplace. (Gerbert 1987)
22	31	Requirements for enhanced standard precautions when performing aerosol generating procedures will be/has been a personal financial burden.
N/A	32	In my workplace, extra costs for enhanced standard precautions when performing aerosol generating procedures have been charged to patients as extra fees.
N/A	33	My income has been reduced since returning to work during the COVID-19 pandemic

Survey 1 Question Number	Survey 2 Question Number	Question
**Domain 5. Confidence in IC and PPE effectiveness & availability**		
N/A	23	I am confident that the COVID-19 safety and infection control protocols adequately protect oral health care providers.
N/A	24	I am confident that the COVID-19 safety and infection control protocols adequately protect patients.
N/A	25	If there is a second wave of COVID19 in my practice location, I will continue to work and change infection control protocols as required. (Naghavi et al. 2012)
20	N/A	There will be an adequate supply of Personal Protective Equipment available to protect me from COVID-19. (Jin et al. 2020)
N/A	27	My preferred PPE items are available to purchase when I need them. (Jin et al. 2020)
N/A	28	The availability of certain PPE items influences my choice of PPE.
**Domain 6. IPC Protocols and PPE use practices**		
N/A	34	Before the COVID-19 pandemic, I always wore a mask preoperatively/during a medical history.
N/A	35	During the COVID-19 pandemic, I always wear a mask preoperatively/during a medical history.
N/A	36	Before the COVID-19 pandemic, I knew and strictly followed the procedures for donning protective equipment. (Dost et al. 2020)
N/A	38	During the COVID-19 pandemic, I know and strictly follow the procedures for donning protective equipment. (Dost et al. 2020)
N/A	37	Before the COVID-19 pandemic, I knew and strictly followed the procedures for doffing protective equipment. (Dost et al. 2020)
N/A	39	During the COVID-19 pandemic, I know and strictly follow the procedures for doffing protective equipment. (Dost et al. 2020)
N/A	40	During the COVID 19 pandemic, my coworkers appear to know the correct PPE to wear and strictly follow the procedures for donning and doffing protective equipment.
N/A	41	I observe a settling time after aerosol generating procedures before disinfection of the operatory.
N/A	42	I perform aerosol generating procedures only in enclosed operatories.

Appendix Table 2. Perceived Risks Upon Returning to Practice.
☐ survey 1 question, ☐ Survey 2 question, ☐ Survey 1 and 2 question. *Comparisons made using Chi-square analysis (or Fisher's exact test when more than 20) of cells contained expected frequencies of less than 5) for between professions comparisons; McNemar's test for comparisons between surveys; all statistical tests were two-tailed. Significant results indicated in red font.

Survey Question		Dentist				RDA				RDH				comparison between professions*	
		Survey 1 (N=246)		Survey 2 (N=182)		Survey 1 (N=363)		Survey 2 (N=253)		Survey 1 (N=270)		Survey 2 (N=135)		Survey 1	Survey 2
		n	%	n	%	n	%	n	%	n	%	n	%		
Dental/DH/DA procedures will increase/have increased my risk for COVID-19.	SA/A	120	(48.8)	99	(55.0)	304	(83.7)	198	(78.6)	238	(88.1)	119	(88.8)	<0.001	<0.001
	N	47	(19.1)	29	(16.1)	37	(10.2)	36	(14.3)	23	(8.5)	8	(6.0)		
	D/SD	79	(32.1)	52	(28.9)	22	(6.1)	18	(7.1)	9	(3.3)	7	(5.2)		
comparison between surveys*		0.437				0.24				0.456					
My family will be/is at greater risk because of my work.	SA/A	111	(45.7)	94	(51.9)	280	(78.0)	191	(76.4)	229	(85.1)	107	(79.9)	<0.001	<0.001
	N	45	(18.5)	31	(17.1)	50	(13.9)	39	(15.6)	20	(7.4)	15	(11.2)		
	D/SD	87	(35.8)	56	(30.9)	29	(8.1)	20	(8.0)	20	(7.4)	12	(9.0)		
comparison between surveys*		0.423				0.847				0.365					
Family/friends will/now distance themselves from me because of my work.	SA/A	40	(16.4)	15	(8.5)	172	(47.5)	52	(21.2)	132	(49.6)	23	(17.4)	<0.001	<0.001
	N	52	(21.3)	19	(10.8)	107	(29.6)	73	(29.8)	74	(27.8)	41	(31.1)		
	D/SD	152	(62.3)	142	(80.7)	83	(22.9)	120	(49.0)	60	(22.6)	68	(51.5)		
comparison between surveys*		<0.001				<0.001				<0.001					
Patients will be reluctant to continue in my care due to the perceived "high-risk" environment created by dental procedures.	SA/A	66	(27.0)	--	--	103	(29.0)	--	--	119	(44.6)	--	--	<0.001	N/A
	N	57	(23.4)	--	--	151	(42.5)	--	--	88	(33.0)	--	--		
	D/SD	121	(49.6)	--	--	101	(28.5)	--	--	60	(22.5)	--	--		
comparison between surveys*		N/A				N/A				N/A					
Though my office is open to provide comprehensive care, I notice a decrease in patient requests for routine treatment appointments.	SA/A	--	--	33	(19.1)	--	--	38	(15.8)	--	--	11	(8.7)	N/A	0.099
	N	--	--	32	(18.5)	--	--	58	(24.2)	--	--	28	(22.2)		
	D/SD	--	--	108	(62.4)	--	--	144	(60.0)	--	--	87	(69.0)		
comparison between surveys*		N/A				N/A				N/A					
I have heard patients express concerns about COVID19 in the dental office.	SA/A	--	--	79	(45.1)	--	--	158	(63.7)	--	--	88	(67.7)	N/A	<0.001
	N	--	--	20	(11.4)	--	--	42	(16.9)	--	--	16	(12.3)		
	D/SD	--	--	76	(43.4)	--	--	48	(19.4)	--	--	26	(20.0)		
comparison between surveys*		N/A				N/A				N/A					
There is a reduced presence of escorts, parents and guardians in the operatory room.	SA/A	--	--	161	(91.0)	--	--	204	(82.9)	--	--	105	(81.4)	N/A	0.107
	N	--	--	7	(4.0)	--	--	16	(6.5)	--	--	11	(8.5)		
	D/SD	--	--	9	(5.1)	--	--	26	(10.6)	--	--	13	(10.1)		
comparison between surveys*		N/A				N/A				N/A					
Media and news coverage related to COVID-19 is stressful.	SA/A	199	(80.9)	--	--	268	(74.9)	--	--	210	(78.1)	--	--	0.316	N/A
	N	30	(12.2)	--	--	57	(15.9)	--	--	43	(16.0)	--	--		
	D/SD	17	(6.9)	--	--	33	(9.2)	--	--	16	(5.9)	--	--		
comparison between surveys*		N/A				N/A				N/A					

Appendix Table 3. Workplace Preparedness for Returning to Practice.

☐ survey 1 question, ☐ Survey 2 question, ☐ Survey 1 and 2 question. *Comparisons made using Chi-square analysis (or Fisher's exact test when more than 20) of cells contained expected frequencies of less than 5) for between professions comparisons; McNemar's test for comparisons between surveys; all statistical tests were two-tailed. Significant results indicated in red font.

Survey Question		Dentist				RDA				RDH				comparison between professions*	
		Survey 1 (N=246)		Survey 2 (N=182)		Survey 1 (N=363)		Survey 2 (N=253)		Survey 1 (N=270)		Survey 2 (N=135)		Survey 1	Survey 2
		n	%	n	%	n	%	n	%	n	%	n	%		
Return to Work Guidelines for the Oral Health Professions of Nova Scotia are/have been helpful in guiding return to practice.	SA/A	226	(91.9)	173	(96.1)	276	(76.5)	210	(84.0)	214	(79.6)	116	(87.2)	<0.001	0.003
	N	12	(4.9)	7	(3.9)	54	(15.0)	32	(12.8)	31	(11.5)	14	(10.5)		
	D/SD	8	(3.3)	0	(0.0)	31	(8.6)	8	(3.2)	24	(8.9)	3	(2.3)		
comparison between surveys*		0.043				0.016				0.037					
My workplace has developed its site-specific reopening plan based on the Emergency and Urgent Care "Return to Work Guidelines for the Oral Health Professions of Nova Scotia".	SA/A	234	(96.7)	--	--	296	(84.3)	--	--	222	(85.1)	--	--	<0.001	N/A
	N	8	(3.3)	--	--	31	(8.8)	--	--	26	(10.0)	--	--		
	D/SD	0	(0.0)	--	--	24	(6.8)	--	--	13	(5.0)	--	--		
comparison between surveys*		N/A				N/A				N/A					
Protocols and guidelines for screening and managing suspected/confirmed COVID-19 patients have been developed at my workplace.	SA/A	239	(98.0)	176	(97.2)	330	(92.7)	235	(94.0)	233	(90.0)	129	(97.0)	0.002	0.282
	N	3	(1.2)	3	(1.7)	8	(2.2)	6	(2.4)	14	(5.4)	3	(2.3)		
	D/SD	2	(0.8)	2	(1.1)	18	(5.1)	9	(3.6)	12	(4.6)	1	(0.8)		
comparison between surveys*		0.891				0.691				0.039					
The initial preparation at my workplace was enough to prevent the spread of COVID-19.	SA/A	--	--	145	(81.0)	--	--	155	(61.8)	--	--	85	(65.4)	N/A	<0.001
	N	--	--	25	(14.0)	--	--	75	(29.9)	--	--	27	(20.8)		
	D/SD	--	--	9	(5.0)	--	--	21	(8.4)	--	--	18	(13.8)		
comparison between surveys*		N/A				N/A				N/A					
Before scheduling appointments in my workplace, patients are pre-screened using remote communications for COVID-19 symptoms, risk factors, underlying medical risk factors and the nature of the chief complaint.	SA/A	--	--	178	(98.3)	--	--	237	(95.6)	--	--	124	(93.9)	N/A	0.262
	N	--	--	2	(1.1)	--	--	6	(2.4)	--	--	3	(2.3)		
	D/SD	--	--	1	(0.6)	--	--	5	(2.0)	--	--	5	(3.8)		
comparison between surveys*		N/A				N/A				N/A					
Before allowing patients entry into the clinic, staff rescreen them for COVID-19 symptoms, risk factors, underlying medical risk factors and the nature of the chief complaint.	SA/A	--	--	175	(97.2)	--	--	228	(91.2)	--	--	117	(89.3)	N/A	0.075
	N	--	--	2	(1.1)	--	--	9	(3.6)	--	--	6	(4.6)		
	D/SD	--	--	3	(1.7)	--	--	13	(5.2)	--	--	8	(6.1)		
comparison between surveys*		N/A				N/A				N/A					

Appendix Table 4. Individual Preparedness for Returning to Practice

☐ survey 1 question, ☐ Survey 2 question, ☐ Survey 1 and 2 question. *Comparisons made using Chi-square analysis (or Fisher's exact test when more than 20) of cells contained expected frequencies of less than 5) for between professions comparisons; McNemar's test for comparisons between surveys; all statistical tests were two-tailed. Significant results indicated in red font.

Survey Question		Dentist				RDA				RDH				comparison between professions*	
		Survey 1 (N=246)		Survey 2 (N=182)		Survey 1 (N=363)		Survey 2 (N=253)		Survey 1 (N=270)		Survey 2 (N=135)		Survey 1	Survey 2
		n	%	n	%	n	%	n	%	n	%	n	%		
I am comfortable with my understanding of COVID-19 symptoms and risk factors.	SA/A	243	(99.2)	178	(98.3)	328	(90.9)	228	(90.1)	248	(91.9)	124	(93.2)	<0.001	0.007
	N	2	(0.8)	1	(0.6)	17	(4.7)	17	(6.7)	15	(5.6)	4	(3.0)		
	D/SD	0	(0.0)	2	(1.1)	16	(4.4)	8	(3.2)	7	(2.6)	5	(3.8)		
comparison between surveys*		0.244				0.426				0.438					
I am comfortable with the education and training I have received on COVID-19 safety and infection control protocols.	SA/A	231	(93.9)	175	(96.7)	246	(69.1)	200	(79.1)	181	(69.3)	112	(84.8)	<0.001	<0.001
	N	9	(3.7)	4	(2.2)	61	(17.1)	34	(13.4)	46	(17.6)	10	(7.6)		
	D/SD	6	(2.4)	2	(1.1)	49	(13.8)	19	(7.5)	34	(13.0)	10	(7.6)		
comparison between surveys*		0.408				0.014				0.003					
I am comfortable with my understanding of safety and infection control protocols.	SA/A	242	98.8)	179	(98.9)	316	(87.3)	232	(92.1)	235	(87.7)	124	(92.5)	<0.001	0.027
	N	1	(0.4)	0	(0.0)	27	(7.5)	11	(4.4)	18	(6.7)	5	(3.7)		
	D/SD	2	(0.8)	2	(1.1)	19	(5.2)	9	(3.6)	15	(5.6)	5	(3.7)		
comparison between surveys*		0.660				0.166				0.324					
I have the skills I need to effectively treat patients during the COVID-19 pandemic.	SA/A	239	(98.0)	169	(93.4)	285	(80.5)	211	(84.1)	209	(79.5)	111	(84.1)	<0.001	0.033
	N	3	(1.2)	9	(5.0)	55	(15.5)	34	(13.5)	38	(14.4)	16	(12.1)		
	D/SD	2	(0.8)	3	(1.7)	14	(4.0)	6	(2.4)	16	(6.1)	5	(3.8)		
comparison between surveys*		0.050				0.425				0.484					
I will get a vaccine to protect against COVID-19 when one becomes available.	SA/A	216	(89.3)	163	(90.6)	284	(79.1)	196	(78.1)	228	(85.1)	109	(81.3)	0.009	0.01
	N	14	(5.8)	11	(6.1)	53	(14.8)	44	(17.5)	28	(10.4)	21	(15.7)		
	D/SD	12	(5.0)	6	(3.3)	22	(6.1)	11	(4.4)	12	(4.5)	4	(3.0)		
comparison between surveys*		0.713				0.458				0.266					

Appendix Table 5. Financial Concerns About Practicing During COVID-19 Pandemic.

☐ survey 1 question, ☐ Survey 2 question, ☐ Survey 1 and 2 question. *Comparisons made using Chi-square analysis (or Fisher's exact test when more than 20) of cells contained expected frequencies of less than 5) for between professions comparisons; McNemar's test for comparisons between surveys; all statistical tests were two-tailed. Significant results indicated in red font.

Survey Question		Dentist				RDA				RDH				comparison between professions	
		Survey 1 (N=246)		Survey 2 (N=182)		Survey 1 (N=363)		Survey 2 (N=253)		Survey 1 (N=270)		Survey 2 (N=135)		Survey 1	Survey 2
		n	%	n	%	n	%	n	%	n	%	n	%		
The additional cost of enhanced standard precautions when performing aerosol generating procedures influences my choice of PPE	SA/A	--	--	77	(44.0)	--	--	74	(32.7)	--	--	31	(26.5)	N/A	<0.001
	N	--	--	29	(16.6)	--	--	74	(32.7)	--	--	27	(23.1)		
	D/SD	--	--	69	(39.4)	--	--	78	3(4.5)	--	--	59	(50.4)		
comparison between surveys*		NA				NA				NA					
Requirements for enhanced standard precautions when performing aerosol generating procedures will be/has been a significant financial burden to my office/workplace.	SA/A	214	(89.2)	127	(72.2)	203	(60.2)	107	(50.5)	179	(69.6)	71	(63.4)	<0.001	<0.001
	N	22	(9.2)	33	(18.8)	108	(32.0)	79	(37.3)	62	(24.1)	22	(19.6)		
	D/SD	4	(1.7)	16	(9.1)	26	(7.7)	26	(12.3)	16	(6.2)	19	(17.0)		
comparison between surveys*		<0.001				0.048				0.005					
Requirements for enhanced standard precautions when performing aerosol generating procedures will be/has been a personal financial burden.	SA/A	188	(78.0)	107	(62.9)	62	(20.1)	23	(11.3)	55	(22.3)	7	(6.5)	<0.001	<0.001
	N	27	(11.2)	27	(15.9)	98	(31.8)	54	(26.6)	55	(22.3)	16	(14.8)		
	D/SD	26	(10.8)	36	(21.2)	148	(48.1)	126	(62.1)	137	(55.5)	85	(78.7)		
comparison between surveys*		0.003				0.004				<0.001					
In my workplace, extra costs for enhanced standard precautions when performing aerosol generating procedures have been charged to patients as extra fees.	SA/A	--	--	64	(36.0)	--	--	83	(37.6)	--	--	57	(47.5)	N/A	<0.001
	N	--	--	11	(6.2)	--	--	33	(14.9)	--	--	2	(1.7)		
	D/SD	--	--	103	(57.9)	--	--	105	(47.5)	--	--	61	(50.8)		
comparison between surveys*		NA				NA				NA					
My income has been reduced since returning to work during the COVID-19 pandemic	SA/A	--	--	135	(75.8)	--	--	35	(15.4)	--	--	23	(18.4)	N/A	<0.001
	N	--	--	24	(13.5)	--	--	26	(11.4)	--	--	9	(7.2)		
	D/SD	--	--	19	(10.7)	--	--	167	(73.2)	--	--	93	(74.4)		
comparison between surveys*		NA				NA				NA					

Appendix Table 6. Confidence in IC and PPE effectiveness & availability.

☐ survey 1 question, ☐ Survey 2 question, ☐ Survey 1 and 2 question. *Comparisons made using Chi-square analysis (or Fisher's exact test when more than 20) of cells contained expected frequencies of less than 5) for between professions comparisons; McNemar's test for comparisons between surveys; all statistical tests were two-tailed. Significant results indicated in red font.

Survey Question		Dentist				RDA				RDH				comparison between professions*	
		Survey 1 (N=246)		Survey 2 (N=182)		Survey 1 (N=363)		Survey 2 (N=253)		Survey 1 (N=270)		Survey 2 (N=135)		Survey 1	Survey 2
		n	%	n	%	n	%	n	%	n	%	n	%		
I am confident that the COVID-19 safety and infection control protocols adequately protect oral health care providers.	SA/A	--	--	164	(90.6)	--	--	148	(58.7)	--	--	77	(58.3)	N/A	<0.001
	N	--	--	13	(7.2)	--	--	66	(26.2)	--	--	33	(25.0)		
	D/SD	--	--	4	(2.2)	--	--	38	(15.1)	--	--	22	(16.7)		
comparison between surveys*		N/A				N/A				N/A					
I am confident that the COVID-19 safety and infection control protocols adequately protect patients.	SA/A	--	--	173	(95.6)	--	--	180	(71.7)	--	--	92	(69.7)	N/A	<0.001
	N	--	--	3	(1.7)	--	--	48	(19.1)	--	--	24	(18.2)		
	D/SD	--	--	5	(2.8)	--	--	23	(9.2)	--	--	16	(12.1)		
comparison between surveys*		N/A				N/A				N/A					
There will be an adequate supply of Personal Protective Equipment available to protect me from COVID-19.	SA/A	103	(42.4)	--	--	167	(46.6)	--	--	89	(33.8)	--	--	<0.004	N/A
	N	58	(23.9)	--	--	99	(27.7)	--	--	92	(35.0)	--	--		
	D/SD	82	(33.7)	--	--	92	(25.7)	--	--	82	(31.2)	--	--		
comparison between surveys*		N/A				N/A				N/A					
My preferred PPE items are available to purchase when I need them.	SA/A	--	--	92	(53.5)	--	--	128	(54.5)	--	--	60	(47.2)	N/A	0.175
	N	--	--	24	(14.0)	--	--	44	(18.7)	--	--	31	(24.4)		
	D/SD	--	--	56	(32.6)	--	--	63	(26.8)	--	--	36	(28.3)		
comparison between surveys*		N/A				N/A				N/A					
The availability of certain PPE items influences my choice of PPE.	SA/A	--	--	132	(75.4)	--	--	134	(56.3)	--	--	85	(66.9)	N/A	<0.001
	N	--	--	20	(11.4)	--	--	65	(27.3)	--	--	26	(20.5)		
	D/SD	--	--	23	(13.1)	--	--	39	(16.4)	--	--	16	(12.6)		
comparison between surveys*		N/A				N/A				N/A					

Appendix Table 7. Infection and Prevention Control (IPC) Protocols and Personal Protective Equipment (PPE) Use Practices.

☐ survey 1 question, ☐ Survey 2 question, ☐ Survey 1 and 2 question. *Comparisons made using Chi-square analysis (or Fisher's exact test when more than 20) of cells contained expected frequencies of less than 5) for between professions comparisons; McNemar's test for comparisons between surveys; all statistical tests were two-tailed. Significant results indicated in red font.

Survey Question		Dentist				RDA				RDH				comparison between professions*	
		Survey 1 (N=246)		Survey 2 (N=182)		Survey 1 (N=363)		Survey 2 (N=253)		Survey 1 (N=270)		Survey 2 (N=135)		Survey 1	Survey 2
		n	%	n	%	n	%	n	%	n	%	n	%		
Before the COVID-19 pandemic, I always wore a mask preoperatively/during a medical history.	SA/A	--	--	49	(27.4)	--	--	85	(34.3)	--	--	22	(16.5)	N/A	**<0.001**
	N	--	--	10	(5.6)	--	--	23	(9.3)	--	--	3	(2.3)		
	D/SD	--	--	120	(67.0)	--	--	140	(56.5)	--	--	108	(81.2)		
comparison between surveys*		N/A				N/A				N/A					
During the COVID-19 pandemic, I always wear a mask preoperatively/during a medical history.	SA/A	--	--	175	(98.3)	--	--	238	(96.4)	--	--	123	(95.3)	N/A	0.583
	N	--	--	0	(0.0)	--	--	2	(0.8)	--	--	1	(0.8)		
	D/SD	--	--	3	(1.7)	--	--	7	(2.8)	--	--	5	(3.9)		
comparison between surveys*		N/A				N/A				N/A					
Before the COVID-19 pandemic, I knew and strictly followed the procedures for donning protective equipment.	SA/A	--	--	97	(54.5)	--	--	167	(69.3)	--	--	85	(63.9)	N/A	**0.036**
	N	--	--	31	(17.4)	--	--	30	(12.4)	--	--	16	(12.0)		
	D/SD	--	--	50	(28.1)	--	--	44	(18.3)	--	--	32	(24.1)		
comparison between surveys*		N/A				N/A				N/A					
During the COVID-19 pandemic, I know and strictly follow the procedures for donning protective equipment.	SA/A	--	--	174	(98.3)	--	--	236	(96.3)	--	--	130	(98.5)	N/A	0.484
	N	--	--	2	(1.1)	--	--	5	(2.0)	--	--	2	(1.5)		
	D/SD	--	--	1	(0.6)	--	--	4	(1.6)	--	--	0	(0.0)		
comparison between surveys*		N/A				N/A				N/A					
Before the COVID-19 pandemic, I knew and strictly followed the procedures for doffing protective equipment.	SA/A	--	--	99	(55.6)	--	--	160	(66.7)	--	--	90	(67.7)	N/A	0.011
	N	--	--	28	(15.7)	--	--	39	(16.3)	--	--	11	(8.3)		
	D/SD	--	--	51	(28.7)	--	--	41	(17.1)	--	--	32	(24.1)		
comparison between surveys*		N/A				N/A				N/A					
During the COVID-19 pandemic, I know and strictly follow the procedures for doffing protective equipment.	SA/A	--	--	175	(98.3)	--	--	236	(95.9)	--	--	127	(96.9)	N/A	0.683
	N	--	--	2	(1.1)	--	--	6	(2.4)	--	--	3	(2.3)		
	D/SD	--	--	1	(0.6)	--	--	4	(1.6)	--	--	1	(0.8)		
comparison between surveys*		N/A				N/A				N/A					
During the COVID-19 pandemic, my coworkers appear to know the correct PPE to wear and strictly follow the procedures for donning and doffing protective equipment.	SA/A	--	--	176	(98.3)	--	--	211	(84.7)	--	--	101	(78.3)	N/A	**<0.001**
	N	--	--	1	(0.6)	--	--	19	(7.6)	--	--	13	(10.1)		
	D/SD	--	--	2	(1.1)	--	--	19	(7.6)	--	--	15	(11.6)		
comparison between surveys*		N/A				N/A				N/A					

Survey Question		Dentist				RDA				RDH				comparison between professions*	
		Survey 1 (N=246)		Survey 2 (N=182)		Survey 1 (N=363)		Survey 2 (N=253)		Survey 1 (N=270)		Survey 2 (N=135)		Survey 1	Survey 2
		n	%	n	%	n	%	n	%	n	%	n	%		
I observe a settling time after aerosol generating procedures before disinfection of the operatory.	SA/A	--	--	32	(18.4)	--	--	55	(22.5)	--	--	19	(15.1)	N/A	0.395
	N	--	--	26	(14.9)	--	--	40	(16.4)	--	--	18	(14.3)		
	D/SD	--	--	116	(66.7)	--	--	149	(61.1)	--	--	89	(70.6)		
comparison between surveys*		N/A				N/A				N/A					
I perform aerosol generating procedures only in enclosed operatories.	SA/A	--	--	55	(31.4)	--	--	63	(26.0)	--	--	35	(29.4)	N/A	0.131
	N	--	--	7	(4.0)	--	--	24	(9.9)	--	--	13	(10.9)		
	D/SD	--	--	113	(64.6)	--	--	155	(64.0)	--	--	71	(59.7)		
comparison between surveys*		N/A				N/A				N/A					
